# Approach to large thrombus burden in ST-elevation myocardial infarction

**DOI:** 10.3389/fcvm.2025.1610917

**Published:** 2026-02-09

**Authors:** Rohit Mody, Debabrata Dash, Bhavya Mody, Umanshi Dash, Rajeev Gupta

**Affiliations:** 1 Department of Cardiology, Mody Harvard Cardiac Institute & Research Centre, Krishna Super Specialty Hospital, Bathinda, India; 2 Department of Cardiology, Aster Hospital, Dubai, United Arab Emirates; 3 Department of Internal Medicine, Resident Doctor, Trinity Health Hospital, Livonia, MI, United States; 4 Department of Cardiology, Spectrum Medical Center and Burjeel Royal Hospital, Al Ain, United Arab Emirates

**Keywords:** distal embolization, high thrombus burden, percutaneous coronary intervention, ST-elevation myocardial infarction, thrombectomy

## Abstract

In ST-Elevation Myocardial Infarction (STEMI), Primary Percutaneous Coronary Intervention (PPCI) is the preferred treatment. However, High Thrombus Burden (HTB), defined by extensive thrombus in the Infarct-Related Artery (IRA), particularly with “cut-off” occlusion patterns and large vessel diameter (>3.5 mm), presents a major challenge. Despite the use of effective anticoagulation, glycoprotein IIb/IIIa inhibitors, and Dual Antiplatelet Therapy (DAPT), HTB remains a significant obstacle. It is associated with complications such as no/slow reflow and distal embolization, leading to adverse cardiovascular outcomes. Accurate assessment using angiographic features and MI thrombus grading is essential before choosing a therapeutic strategy. Management options include mechanical thrombectomy, aspiration, parenteral/oral antiplatelets, intracoronary thrombolytics, and stenting approaches like direct stenting. Success depends on tailored, scenario-specific application of these therapies. This review highlights current strategies to manage HTB during PPCI.

## 1 Introduction

Acute ST-Elevation Myocardial Infarction (STEMI) frequently reported in patients from the disruption of an atherosclerotic plaque, triggering thrombus formation that ultimately occludes the major epicardial coronary arteries (1). Primary Percutaneous Coronary Intervention (PPCI) when contrasted with thrombolytic therapy has emerged as the safest and best treatment approach for STEMI (2–4). Evidence from randomized trials and real-world clinical practice has shown that stenting during PPCI improves both short- and long-term outcomes in comparison to balloon angioplasty alone (5, 6). The introduction of Drug-Eluting Stents (DESs) has further enhanced clinical outcomes in this setting (7, 8). In addition, GPI IIb/IIIa antagonist administration during Percutaneous Coronary Intervention (PCI) has been associated with improved clinical outcomes (9). A high Intracoronary Thrombus (ICT) has emerged as the major risk factor for complications, including the no-reflow phenomenon, stent thrombosis, distal embolization, and adverse cardiovascular events inspite of the availability of potent anticoagulant and anti-platelet regimens, Angiographic evidence of ICT is observed in 91.6% of patients with STEMI (1), with massive thrombus noted in about 15% of those presenting with Acute Coronary Syndrome (ACS) (10). The risk of procedural complications, including distal embolization, abrupt closure, and persistent or transient no-reflow, is significantly increased by the presence of ICT (11). Effectively managing ICT is critical for successful PPCI and involves combining mechanical and pharmacological strategies to address thrombus burden before stent deployment, thereby reducing associated complications. This section discusses the pathogenesis, causes, sequelae, and management strategies for ICT in patients with STEMI.

## 2 Pathophysiology of generation of intracoronary thrombus

In most cases, the erosion or rupture of an underlying atherosclerotic plaque, which exposes the thrombogenic subendothelial matrix to circulating platelets, is the main cause of thrombus formation (12). Following plaque disruption, acute coronary thrombosis develops in approximately 70% of the cases, whereas plaque erosion accounts for the remaining 30% (13). Plaque erosion is more frequently observed in females, patients with hypertriglyceridemia, and those with diabetes. Plaques with a thin fibrous cap and a large lipid core are particularly prone to disruption or erosion due to infiltration by lipid-filled macrophages (foam cells) (14).

The coagulation cascade is activated by atherosclerotic plaque rupture through two synergistic pathways. In the first stage, direct binding of platelet GPI VI with collagen exposed by the denyded endothelium is seen. Subsequently, interaction of GPI Ib-V-IX occurred with collagen-bound von Willebrand Factor (vWF), potentiating activation, binding and aggregation of platelet, thereby leading to formation of white thrombus. The second pathway involves the tissue factor, which initiates a proteolytic cascade, thereby causing thrombin production. Thrombin converts fibrinogen to fibrin, releasing agonists such as adenosine, serotonin, and thromboxane A2, which further stimulate platelet activation and aggregation. Thrombus formation is amplified by this cascade. Intracoronary thrombi typically comprise inflammatory cells, fibrin, and erythrocytes, eventually forming dense fibrin-rich thrombi that are difficult to disrupt mechanically or pharmacologically over time (15–17).

**Figure 1:**
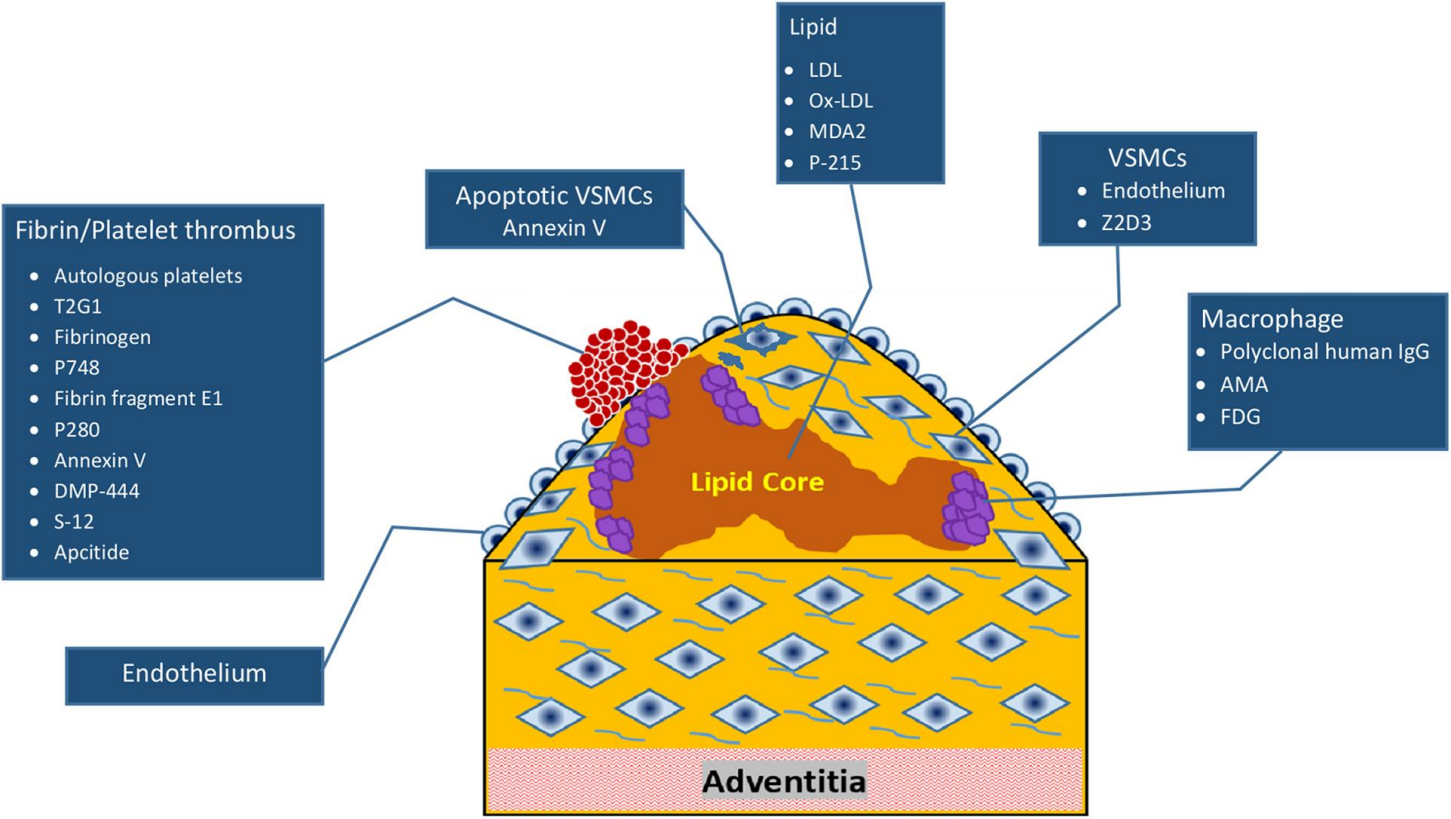
Pathophysiology of intracoronary thrombus formation. LDL, low-density lipoprotein; VSMC, vascular smooth muscle cell; AMA, alternative macrophage activation; FDG, fluorodeoxyglucose.

The friability of a thrombus influences its susceptibility to mechanical intervention during PCI. Late-presenting thrombi frequently exhibit resistance to aspiration catheters, balloons, and other coronary devices owing to thrombus variability. Thrombi comprise two types of fibrin fibers: thin dense fibers that are more resistant to dissolution by mechanical devices and thrombolytics, and thick fibrin fibers that are even more challenging to manage. The pathophysiology of ICT formation is illustrated in Figure 1.

### 2.1 Quantification of coronary thrombi

In patients with STEMI, grading tools are frequently employed for quantifying the ICT burden, with angiography playing a crucial role in its assessment. Angiographically, ICT is characterized by the identification of a lack of filling accompanied by declined density of contrast or cloudiness. The extensively utilized grading tool is the Thrombolysis in Myocardial Infarction (TIMI) scale, which evaluates the thrombus relative size compared with the affected vessel. This scale ranges from grade 0, indicating the absence of a thrombus, to grade 5, representing a massive thrombus causing complete vessel occlusion.

Grade 0 indicates the absence of thrombus or any angiographic filling defect. Grade 1 denotes the possibility of a thrombus, as indicated by angiographic signs, including irregular lesion contours, smooth convex meniscus, or reduced contrast density at the site of occlusion. Grade 2 indicates a definite thrombus with dimensions ≤50% diameter of vessel. Grade 3 represents a definite thrombus with length exceeding 50% the vessel diameter. Grade 4 indicates a large thrombus with dimensions greater than two vessel diameters. Grade 5 signifies a very large thrombus causing complete vessel occlusion (18).

Sianos et al. suggested that when a grade 5 thrombus is present, a 1.25- or 1.5 mm balloon or a guidewire should be used to recanalize the artery. Residual thrombus is further classified into grade 0, indicating no residual thrombus; grades 1–3, representing small residual thrombi; and grades 4–5, indicating large residual thrombi. Nicoli et al. proposed a simplified classification wherein TIMI grades 1–3 are considered low grade, whereas TIMI grades 4–5 are categorized as high grade. This simplified system provides a practical approach to thrombus evaluation while retaining clinical significance (19, 20).

### 2.2 Angiographic indicators of large thrombus burden

#### 2.2.1 Angiographic indicators of large thrombus burden

Yip et al. identified six angiographic features suggestive of significant thrombogenesis. These features are as follows: (a) an occlusion cutoff pattern, (b) an accumulated thrombus proximal to the occlusion, (c) an IRA reference lumen diameter exceeding 4.0 mm, (d) incomplete occlusion characterized by with a thrombus on angiogram, maximum length dimensions greater than 3× lumen diameter for the reference, (e) To the proximal lesion, presence of a floating thrombus and (f) presence of persistent dye stasis distal to the occluded site (21). These indicators collectively aid in identifying and quantifying large thrombus burdens, enabling tailored therapeutic interventions during PPCI. The grading of the thrombus in the angiographic assessments, which provides additional insight into the thrombus burden, is detailed in Table 1.

**Table 1:** TIMI thrombus scale.

Grade no.	Grade description
Grade 0	Angiographic evidence of thrombus is absent.
Grade 1	Possible thrombus: irregular lesions contour, decreased contrast density or haziness, a smooth convex meniscus at the site of a total occlusion suggestive, but not firmly diagnostic of thrombus.
Grade 2	Presence of definite thrombus in multiple angiographic characterized by marked irregular lesion with a filling defect. Greatest dimension of thrombus is <½ vessel diameter.
Grade 3	Appearance of definite thrombus in multiple angiographic views with greatest dimension from >½ to >2 vessel diameter.
Grade 4	Definite large size thrombus present with greatest dimension >2 vessel diameters.
Grade 5	Complete vessel thrombotic occlusion.

TIMI, thrombolysis in myocardial infarction.

### 2.3 Intracoronary thrombi and clinical outcomes

The presence of ICT during ACS is correlated with suboptimal coronary reperfusion and worse clinical outcomes (1, 22, 23). Quantifying the impact of a thrombus on the microvascular circulation can be assessed using the Myocardial Blush Grade (MBG) or the degree of ST-segment Resolution (STR) (24). MBG, primarily a biomarker for capillary-level perfusion, and STR have both been shown to serve as independent mortality markers (25).

Grading tools provide a standardized approach for assessing thrombus burden and its correlation with clinical outcomes. Moreover, they guide management decisions both before and during PCI. Persistent chest pain, distal vessel occlusion, and electrocardiogram changes can result from ICT presence and distal embolization into the microcirculation. Even when normal epicardial flow is restored, Microvascular Obstruction (MVO) and distal embolization are associated with adverse outcomes, including reduced ventricular function and larger infarct size.

Considering the severe clinical consequences of inadequately resolving thrombus, interventional cardiologists should adopt effective management strategies aimed at ICT management and prevention during STEMI. Lesion characteristics, disease state, and patient-related factors represent the factors contributing to the thrombus burden during ACS, as outlined in Table 2 (26).

**Table 2:** Factors contributing to thrombus burden during ACS (26).

Category	Factors
Factors related to lesion	Acute rupture of plaque and complex plaque morphology
Factors related to PCI	Thrombocytopenia induced by heparin Inappropriate dual antiplatelets Inappropriate anti coagulation Potentiation of formation of thrombus with guide wire, balloon or stent the angry-clot phenomenon
Factors related to patients	Hyperglycemia Leukocytosis Smoking Vasculitis Male sex Hypercoagulability-hypercholesterolemia, hyperhomocystenemia
Disease related factors	Cardiogenic shock Failed thrombolysis Late presentation (>12 h) Established Q-wave MI with ongoing ischemia

PCI, percutaneous coronary intervention; MI, myocardial infarction.

## 3 Management strategies

During PPCI, ICT severity is strongly associated with both clinical outcomes and procedural success. Effectively managing ICT necessitates a systematic multifaceted approach owing to the complexity of intracoronary thrombogenesis. The management encompasses pharmacotherapy to inhibit the coagulation cascade, mechanical techniques to restore blood flow, and, in some cases, thrombus extraction tools to dissolve the thrombus. Theoretically, thrombus extraction may reduce distal embolization, thereby prevent MVO and ensure complete microvascular reperfusion. In patients with STEMI, the current guidelines recommend PPCI as the preferred therapeutic strategy (27). PPCI effectively restores flow and leads to fewer immediate and long-term unfavorable episodes including Major Adverse Cardiac Events (MACE), in comparison to thrombolysis However, managing lesions with large thrombus burdens poses significant challenges during PPCI. To address this issue and effectively reduce thrombus burdens, various mechanical and pharmacological strategies are employed.

## 4 Pharmacological interventions

Early and effective therapeutic interventions are quite crucial with the objective to influence the self-progressive nature of ICT formation. The early initiation of DAPT significantly reduces the ICT burden and improves the clinical outcomes in patients with STEMI.

Upon STEMI diagnosis, a loading dose of aspirin (as per institutional practice) combined with a P2Y12 antagonist, such as clopidogrel, prasugrel, or ticagrelor, should be promptly administered Aspirin (28), orally or intravenously administered in 150–300 mg doses, inhibits thromboxane A2-mediated platelet aggregation. It exhibits its pharmacological action within 30–60 min and lasts for the lifespan of the platelet. Furthermore, in comparison to other anti-platelet drugs, such as clopidogrel, prasugrel, and ticagrelor, aspirin provides superior clinical outcomes due to its higher potency, rapid onset of action, and efficacy (29).

For patients undergoing PCI, intravenous (IV) P2Y12 inhibitors, including cangrelor, may be recommended in specific scenarios, such as intubation, the absence of prior oral P2Y12 receptor inhibitor treatment, or the inability to take oral agents. This approach ensures optimal platelet inhibition and mitigates High Thrombus Burden (HTB)-associated risks in patients with STEMI (30).

### 4.1 GPI IIb/IIIa inhibitors

GPIs, including abciximab, tirofiban, and eptifibatide, inhibit platelet aggregation by competing with fibrinogen and vWF in binding to the GPIIb/IIIa receptor. These agents are more potent than P2Y12 inhibitors as they halt the final common pathway to platelet aggregation and lower the response of platelets to all agonists, causing rapid and comprehensive inhibition (31). Studies on patients with STEMI have reported that GPIs provide various advantages, including survival benefits and improvements in TIMI flow and thrombus resolution (9, 32). However, these favorable clinical outcomes were predominantly noted in the period preceding significant advancements, such as thrombectomy, contemporary stents, potent P2Y12 inhibitors, and routine DAPT (33). Despite their efficacy in enhancing TIMI flow and reducing thrombus burden, the high bleeding risk associated with GPIs represents a significant limitation.

A meta-analysis of 10,123 patients undergoing PPCI reported that GPIs reduced the incidence of nonfatal MI from 8.3% to 5.1% at the 30-day follow-up; however, this reduction was accompanied by an increased risk of thrombocytopenia and minor bleeding (34).

GPIs routine clinical use of GPIs in STEM1 undergoing PCI is not recommended. Instead, recent guidelines have suggest that GPIs should be reserved for bailout therapy or high-risk patients transferred for PPCI (30).

### 4.2 Intracoronary GP IIb/IIIa

In the IRA, intracoronary abciximab administration theoretically provides an advantage over the IV route by delivering a higher local concentration of the drug, subsequently resulting in greater receptor occupancy, thereby promoting more effective thrombus resolution (35). Small-scale studies have demonstrated the clinical benefits of intracoronary abciximab bolus administration; however, large-scale clinical trials have not corroborated these findings (36).

At the follow-up of 90 days, no significant difference was observed in the composite primary endpoints of abciximab in patients presenting with STEMI, as shown in the AIDA-STEMI trial, which compared the clinical efficacy of intracoronary vs. IV bolus administration. This lack of significant clinical benefit suggests that although intracoronary administration holds theoretical promise, its role remains uncertain (37, 38).

Considering the current evidence, large-scale randomized trials should be conducted to demonstrate the potential benefits of intracoronary GPIs and determine their precise role in STEMI management (30).

### 4.3 Intracoronary thrombolytic agents

Intracoronary thrombolytic agent administration facilitates the delivery of high drug concentrations directly to the thrombus site, potentially achieving effective thrombolysis. Early anecdotal reports and small series investigating the use of intracoronary thrombolytics led to various randomized controlled trials. These studies assessed endpoints, including microvascular perfusion, left ventricular (LV) function, Major Adverse Cardiovascular Events (MACE), infarct size, and epicardial flow (39–43).

A meta-analysis of these trials suggested that intracoronary thrombolysis was associated with a reduced incidence of in-hospital MACE, no significant bleeding risk, and improved STR (44). Despite these promising results, the available data on intracoronary fibrinolytic use remain inadequate. The precise clinical benefit of thrombolytic agents administered intracoronary remains unclear owing to the heterogeneity in study designs and limited patient populations. Additionally, in several of these studies, potent P2Y12 inhibitors were not utilized, which could have influenced the outcomes.

Currently, two ongoing Phase III trials aim to further clarify the role of intracoronary thrombolytics. Among patients undergoing primary PCI for STEMI and large thrombus burden, intracoronary administration of alteplase was not superior to placebo in reducing the composite primary outcome of MACE at 30 days, MBG 0/1, distal embolization or failure to achieve ≥50% ST-segment resolution. These data do not support the routine administration of this therapy in patients with STEMI undergoing primary PCI (45). RESTORE-MI trial (the Restoring Microcirculatory Perfusion in STEMI trial) is focused on Tenecteplase. This trial is anticipated to provide more definitive evidence regarding the efficacy and safety of intracoronary thrombolytic therapy in STEMI management.

### 4.4 Anticoagulants

Maintaining adequate anticoagulation is essential to effectively inhibit the coagulation cascade and prevent thrombus development. In PPCI, Unfractionated Heparin (UFH), bivalirudin, and enoxaparin are the most frequently administered anticoagulants. However, no placebo-controlled randomized trials that directly evaluate the role of UFH in PPCI have been conducted.

The ATOLL trial compared enoxaparin (0.4 mg/kg IV bolus) with UFH in the context of PPCI and reported that enoxaparin significantly reduced secondary endpoints, including recurrent MI, ACS, and urgent revascularization, without a marked rise in bleeding events. However, no significant difference was observed in the primary endpoint of 30-day MACE or significant bleeding. In contrast, the superior clinical efficacy of the enoxaparin over UFH has been demonstrated by finding of meta-analysis of 23 PCI trials in which 30,966 patients (33% undergoing PPCI), were enrolled. In patients undergoing PPCI, enoxaparin was associated with a marked decline in bleeding complications and a lower incidence of mortality (46).

Based on this evidence, the routine administration of IV enoxaparin during PPCI has been assigned a Class II recommendation in the recent European Society of Cardiology (ESC) guidelines. This classification highlights its role as a preferred anticoagulant option for improving outcomes in patients with STEMI undergoing PPCI.

### 4.5 Vasodilators

During PPCI, vasodilators, including nitroglycerin, nitroprusside, and adenosine, are frequently administered in patients with a HTB to reduce the risk of the no-reflow phenomenon and distal embolization. A guiding catheter, infusion balloon, or infusion catheter are used for the administration of these agents.

Vasodilators used in this context can be classified into the following three categories:

(a) **Vasodilators with primary activity on epicardial coronary arteries:** these agents, including nitroglycerin, cause significant dilation of the large coronary arteries with minimal or no effect on microcirculation (29).
(b) **Vasodilators with mixed activity:** agents, including sodium nitroprusside, dilate both large epicardial arterioles and small resistance vessels, providing a broader effect
(c) **Vasodilators targeting coronary microcirculation:** these agents include adenosine and calcium channel blockers, which primarily dilate the microvasculature and improve perfusion at the capillary level (47).

Selecting the appropriate vasodilator depends on the specific clinical scenario and the desired therapeutic target, highlighting the significance of tailored intervention strategies during PPCI.

## 5 Stenting strategy

During PPCI, a large thrombus burden poses several clinical challenges, including difficulties with stent apposition, achieving optimal final TIMI flow, and accurate stent sizing, all of which increase the risk of stent thrombosis. Although not advocated for routine application, aspiration thrombectomy may be judiciously employed in cases with a significant thrombus burden to facilitate complete vessel visualization and optimize stent sizing (48). Owing to their superior clinical outcomes, DESs are generally recommended for PPCI. MGuard stents have been tested in small trials to further mitigate the risk of thrombus-associated distal embolization. However, these stents have not demonstrated sufficient clinical efficacy to support their routine use in practice. This discrepancy emphasizes the significance of individualized strategies and careful device selection to address the thrombus burden during PCI (49, 50).

### 5.1 Direct stenting

Distal embolization during PPCI in thrombotic lesions can cause adverse events, including obstruction of the distal epicardial arteries or microvasculature. Direct stenting has been proposed as a strategy for reducing the distal embolization incidence by minimizing thrombus manipulation and executing thrombus entrapment behind the stent. DS is a technique in which thrombus and loose plaque material are compressed by the stent to reduce the risk of distal embolization. In comparison to conventional stenting, DS is associated with various positive outcomes like marked improvement in ST-resolution and a decline in no-reflow phenomenon and in-hospital mortality. This treatment modality is widely clinically practiced; however, no formal guideline recommendations for it exist.

In a major proportion of patients presenting with high-grade thrombus and acute STEMI, DS or direct-like stenting can be utilized specifically in cases where the distal landing zone is visualized after guidewire crossing or when the stent can cross (51, 52). However, inadequate stent expansion, late stent malapposition, underestimating the actual vessel size, and difficulties in crossing calcified and tortuous stenosis constitute the limitations of direct stenting, which can increase the risks of stent thrombosis or restenosis.

**Figure 2:**
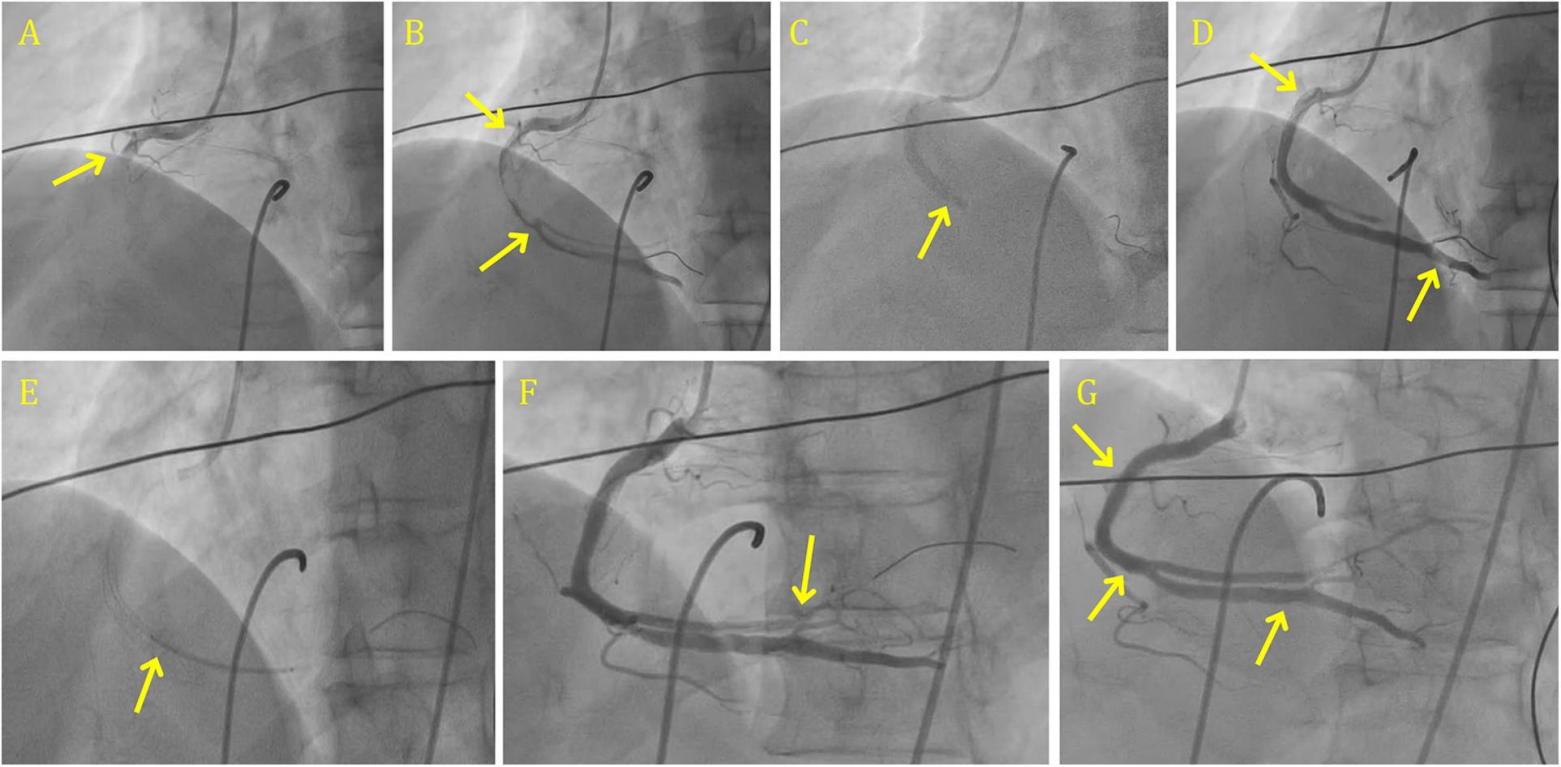
Large thrombus burden angioplasty done by direct stenting in RCA. **(A)** CAG shows 100% occluded RCA with grade 6 clot. **(B)** CAG shows the engagement of RCA after crossing with BMW wire some flow with distal landing zone visible. **(C)** 4.0 × 36 mm DES deployed in the mid RCA by using direct stenting over clot. **(D)** CAG shows the distal RCA and PDA. **(E)** Direct stenting in the PLV using a 2.5 × 38 mm DES over clot. **(F)** CAG showing the persistent clot in the distal PV, over 2.5 mm balloon. **(G)** Final results with TIMI 3 flow and expanded stent. CAG, coronary angiography; RCA, right coronary artery; DES, drug-eluting stent; PDA, patient ductus arteriosus.

A meta-analysis of five RCTs revealed that direct stenting was associated with a reduced incidence of in-hospital cardiovascular death and significant improvements in STR (53) However, considering the absence of definitive clinical benefits in broader contexts, the decision to use direct stenting should be based on lesion anatomy. In carefully selected patients with Acute Myocardial Infarction (AMI) and low-grade thrombus burden, direct stenting, with or without prior Thrombus Aspiration (TA), may be a safe and effective alternative. DS success rates can be increased by using a deflated balloon, as suggested by recent literature. Large thrombus burden angioplasty done by direct stenting is shown in Figure 2.

### 5.2 Summary and practical aspects of direct stenting in thrombus management guideline-directed approach

#### 5.2.1 Direct stenting

- **Rationale:** Seals the thrombus against the vessel wall, minimizing embolization and distal thrombus propagation. Reduces the risk of plaque or thrombus dislodgement compared with balloon predilation.
- **Indications:** HTB (e.g., TIMI thrombus grade ≥4). Soft thrombus or lesions with minimal calcification and low vessel tortuosity.
- **Technique:** Utilizes imaging Intravascular Ultrasonography (IVUS), or Optical Coherence Tomography (OCT) to assess lesion morphology and confirm suitability for direct stenting. Deploys the stent across the thrombotic lesion with gentle inflation to avoid dislodgment.

#### 5.2.2 Anticoagulation and adjunctive pharmacotherapy

- **Agents:** UFH is administered during the procedure to prevent further thrombus formation. GPIs (e.g., abciximab, tirofiban) are administered intracoronary when evidence of ongoing thrombus formation or no-reflow is observed.
- **Guidelines:** Recommended in HTB by the ESC 2023 and ACC/AHA guidelines.

#### 5.2.3 Aspiration thrombectomy (selective use)

- In cases wherein the thrombus is too large to allow safe direct stenting or after failed stent deployment.
- Guidelines discourage routine use but allow selective application in bailout scenarios.

#### 5.2.4 Post-stenting optimization

- High-pressure post-dilation: use a noncompliant balloon for optimizing stent expansion and apposition.
- Intravascular imaging: employ IVUS or OCT for confirming thrombus clearance, assessing stent expansion, and ruling out complications.

### 5.3 Advanced techniques with direct stenting

#### 5.3.1 Pharmacomechanical adjuncts

- Use catheter-directed thrombolysis (e.g., low-dose alteplase) through dedicated devices, such as the AngioJet system, before or immediately after direct stenting for residual thrombus.

#### 5.3.2 Distal embolic protection

- Indications: when treating lesions in high-risk locations, such as saphenous vein grafts (SVGs).
- Deploy a filter-based distal protection device before direct stenting to capture embolic debris.

#### 5.3.3 Staged approach

- In cases of extremely large thrombus burden, consider a deferred stenting strategy:
- Perform aspiration or administer GPIs.
- Delay stent deployment until the thrombus burden is reduced, and flow is restored.

### 5.4 Advantages of direct stenting in large clot burdens

1. Reduced procedure time: avoids multiple steps (e.g., predilation)
2. Lower risk of thrombus embolization: direct sealing of the thrombus prevents distal embolization.
3. Improved outcomes in suitable lesions: studies have suggested better outcomes in lesions with minimal calcification and well-delineated thrombus.

### 5.5 Limitations and risks

#### 5.5.1 Suboptimal stent deployment

Inadequate lesion preparation may lead to incomplete stent expansion or malapposition. A HTB may compromise immediate poststent flow (slow flow or no-reflow).

#### 5.5.2 Distal embolization

Despite care, thrombus migration can occur, particularly if the thrombus is extensive or friable.

#### 5.5.3 Not suitable for all lesions

Tortuous vessels, heavy calcification, or undilatable lesions require predilation.

### 5.6 When not to use direct stenting

• Extremely large thrombus burden with compromised distal flow (TIMI 0–1).

• Calcified lesions requiring vessel preparation if the distal landing zone is not visible.

### 5.7 Summary

In select cases of large thrombus burden, direct stenting represents a viable first-line approach, particularly when the thrombus composition and lesion morphology are favorable. Combining this strategy with guideline-recommended pharmacotherapy and adjunctive mechanical interventions ensures safe and effective thrombus management. Other strategies, including aspiration thrombectomy or pharmacomechanical interventions, can serve as alternatives in cases when direct stenting is inappropriate.

### 5.8 Covered stent

Bare metal stents with a fine mesh covering are used for developing covered stents, enabling thrombus capture and mitigating the risk of distal embolization. Randomized trials have evaluated two types of coronary covered stents, including InspireMD (MGuard) and STENTYS SA.

The MGuard stent can be extended by balloon. It has closed-cell design and is a bare metal stent that is covered with polyethylene terephthalate. Although it has demonstrated benefits, including a reduced mortality rate at 1-year follow-up and complete STR in the MASTER I trial (50), it was associated with significant limitations, such as bulky double-layer design, high stent dislodgment rate, and increased risk of side-branch occlusion. Due to these drawbacks, the MASTER II trial, in which 1114 patients were planned to enrolled, was prematurely discontinued (54).

The STENTYS stent is a self-expanding, self-apposing nitinol platform coated with a drug-containing durable polymer designed to retain the thrombus against the vessel wall. Although its design offers theoretical advantages, including thrombus containment, evidence supporting the routine clinical use of covered stents is currently insufficient (55). To clarify their role in managing thrombotic lesions during PCI, further research is warranted.

### 5.9 Deferred stenting

Deferred stenting is a treatment approach designed to mitigate the risk of no-reflow phenomena and distal embolization by allowing time for partial thrombus resolution and improvement in microvascular dysfunction before stent placement. DEFER-STEMI trial (The Deferred Stenting Versus Immediate Stenting to Prevent No or Slow-Reflow in Acute ST-Segment Elevation Myocardial Infarction trial) enrolled 410 patients with STEMI to compare the outcomes of deferred vs. immediate stenting. The study reported a lower incidence of higher TIMI flow grades and no-flow phenomena at the procedure end in the high-risk population (56). However, in comparison to conventional PCI, at the follow-up of 42-months, no significant reduction was seen in the primary composite endpoint of heart failure, MI, repeat re-vascularization, or all-cause mortality in long-term findings from the DEFER-STEMI trial (57).

Based on the current evidence, the routine clinical use of deferred stenting is not recommended and is categorized as Class III/A. The advantages and disadvantages of deferred stenting compared with direct stenting are detailed in Table 3, providing insights into its potential role in selected clinical scenarios (58).

**Table 3:** The advantages and disadvantages of deferred stenting in comparison to direct stenting.

Advantages	Disadvantages
• Low burden of intracoronary thrombus	• Huge risk of bleeding from extended parenteral anticoagulation
• Larger size of stent	
• Reduced number of stents implantation	• Huge rise in medical expenses
• Smaller size of infarct	• Unplanned revascularization
• Significant thrombolysis in MI flow	• Re-occlusion
• Slow flow/no reflow phenomenon	

MI, myocardial infarction.

## 6 Thrombectomy systems

Thrombectomy devices primarily function to diminish thrombus burdens. These devices are mainly divided into the following two categories: mechanical and manual.

### 6.1 Manual TA

In 1995, Auth et al. introduced the first aspiration catheter system. Subsequent catheter design innovations have yielded substantial improvements, including hydrophilic coatings, enhanced flexibility, tapered distal tips, and lower crossing profiles. Among the manual TA devices, the Export AP remains one of the most frequently employed in clinical practice.

The REMEDIA trial, the first randomized clinical trial to evaluate manual aspiration, demonstrated a significant reduction in the no-reflow phenomenon, distal embolization, and improved STR. However, these improvements did not translate into better clinical outcomes (59). Similarly, the TAPAS trial, which enrolled 1,071 patients, demonstrated a marked decline in MACE, when aspiration manually was utilized alongside PPCI (60). In contrast, subsequent large-scale trials, including TASTE trial (Thrombus aspiration in ST-Elevation Myocardial infarction) TOTAL trial (61) and Thrombectomy with PCI vs. PCI Alone) (62), demonstrated no clinical benefits of routine TA.

**Figure 3:**
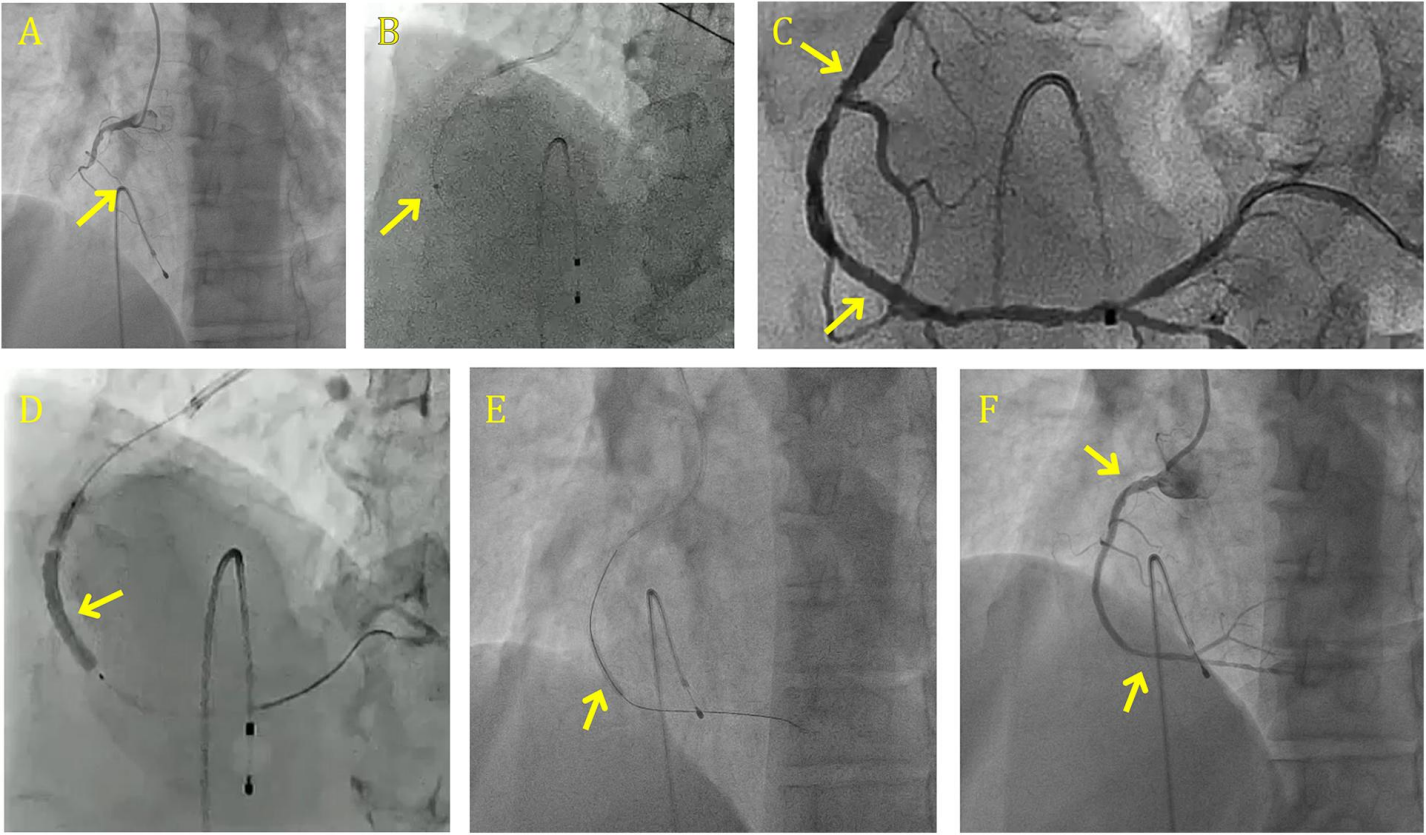
Large thrombus burden angioplasty done aided by manual aspiration catheter. **(A)** CAG shows RCA grade 6 clot with 100% occlusion. **(B)** Angiography showing the manual aspiration first pass in the mid RCA. **(C)** Post-second pass aspiration, a follow-up angiogram was obtained. **(D)** CAG shows the deployment of 3.0 × 40 mm DES in the mid RCA. **(E)** CAG shows the deployment of 3.5 × 30 mm DES in the proximal RCA. **(F)** Good final result with TIMI 3 flow with expanded stent. CAG, coronary angiography; RCA, right coronary artery; DES, drug-eluting stent.

Considering these findings, the 2017 ESC guidelines do not recommend routine TA before PPCI (Class III, Level of Evidence A). although, particular or rescue TA may be considered (Class IIb, Level of Evidence C-LD) in patients who have a massive residual thrombus following vessel following crossing with guidewire and no distal landing zone visible. Large thrombus burden angioplasty done aided by manual aspiration catheter is shown in Figure 3.

Owing to their ease of use and convenience, manual thrombectomy devices provide advantages over motorized equivalents. These devices primarily comprise monorail catheters with a lumen at centre that communicates with one or more openings. Proximally, the attachment of the catheter with a syringe is carried out for manual thrombotic aspiration.

The Diver CE (Invatec), Hunter (IHT Cordynamic), Quick Cat (Spectranetics), Export (Medtronic), and Pronto (Vascular Solutions) are currently available manual thrombectomy devices. Although the operational mechanism of these devices is fundamentally similar, the difference in various aspects like aspiration lumen size, catheter material and theoretical efficiency in thrombus deliverability and extraction is seen. These design variations may influence their performance in clinical practice, providing physicians a variety of options to tailor thrombectomy strategies on the basis of individual patient and lesion characteristics.

(a) Diver CE

The REMEDIA study was the first randomized trial to evaluate a manual thrombectomy device. In this trial, 99 patients undergoing standard PPCI were randomized to either PPCI alone or PPCI with the addition of the Diver CE catheter for manual thrombectomy (63). Findings of study not only demonstrate the safety and efficacy of Diver CE device but also provide evidence that this device leads to marked improvement in STR and MBG.

Furthermore, the risks of slow-reflow, distal embolization, and no-reflow phenomenon were mitigated owing to the device. Subgroup analysis revealed that patients with a HTB or occluded arteries exhibited the most pronounced primary clinical benefits, emphasizing the potential value of manual TA in these specific patient populations.

In patients present with anterior STEMI, more effective STR at 90 min and marked better post-procedure MBG was seen with Diver CE catheter in the findings of another small randomized study (64). These findings confirm the role of manual thrombectomy devices as a valuable adjunct in selected patients with a HTB during PPCI.

(b) Pronto

The DEAR-MI trial evaluated the clinical efficacy of the Pronto catheter in PPCI. In this randomization of 148 patients was done between angioplasty plus thrombectomy/standard angioplasty. The results showed significant improvements in STR in the thrombectomy group, along with a marked reduction in the incidence of the no-reflow phenomenon and distal embolization (65).

Two versions of the Pronto catheter, V3 and low profile, are utilized in PPCI. The LP version features improved deliverability, facilitating effective navigation and treatment of vessels with diameters as small as 1.5 mm. This advantage makes it a versatile tool for managing challenging thrombotic lesions in small and tortuous vessels during PCI.

(c) Export

Several clinical trials have evaluated the clinical efficacy of the Export catheter (66, 67). The EXPIRA trial, which enrolled 175 patients, aimed to assess its effectiveness, focusing on STR and MBG as primary endpoints, with MACEs at the 9-month follow-up as the secondary endpoint (68). The study demonstrated significant improvements in STR at 90 min, better MBG, and reductions in both microvascular damage and infarct size at the 3-month follow-up in patients undergoing manual thrombectomy.

Furthermore, the larger TAPAS trial, a single-center randomized study involving 1,071 patients, utilized the Export catheter. At the follow-up of 1-year, a reduced incidence of cardiac death and nonfatal reinfarction was seen with manual thrombectomy as suggested by the study findings (59). However, LV function or infarct size was not evaluated in the TAPAS trial; instead, it focused on myocardial perfusion improvements. These findings highlight the utility of manual thrombectomy in enhancing myocardial perfusion in patients with STEMI, particularly with the export catheter.

(d) Thrombuster

A recently published retrospective study investigated the efficacy of the Thrombuster thrombectomy device. The findings suggested that, compared with standard PCI, manual thrombectomy using the Thrombuster device led to improved myocardial perfusion and enhanced LV function at the 6-month follow-up (59).

The outcomes of randomized clinical trials comparing standard PCI with PCI plus manual thrombectomy are summarized in Table 4, providing a comprehensive overview of the clinical impact of thrombectomy devices in enhancing the procedural and long-term outcomes.

**Table 4:** Randomized clinical trials to compare standard PCI to PCI plus manual thrombectomy.

Study	Design	No. of patients enrolled	Primary end point	Results
REMEDIA	Single-Centre Study	99	MBG ≥ 2 68% vs 58% (*p* = 0.02) STR ≥ 70%	Results of this prospective randomized study proposed that in comparison to standard PCI, better angiographic outcomes and ECG myocardial reperfusion rate was seen with the manual thrombus-aspiration in unselected patients with STEMI undergoing primary or rescue PCI (55).
De luca et al.	Single-centre study	76	LV remodelling (LV-end diastolic volume at follow-up of 6-months	In patients present with anterior STEMI, reduced incidence of LV remodelling at follow-up of 6 months was reported in patients treated with adjunctive aspiration thrombectomy as compared to conventional stenting (67).
PIHRATE	Single-Centre	DIVER CE	196	In patients of thrombectomy group, marked improvement in the matriculation reperfusion angiographic parameters and ECG STR was seen directly after PCI (71).
DEAR-MI	Single-centre	Pronto	148	In comparison to standard PPCI, better myocardial perfusion was reported in patients of group Manual thrombus aspiration before PPCI (67).
Noel et.al	Multi-centre	Export	50	Trend towards significant improvement in myocardial perfusion and lower incidence of clinical events in patients treated with thrombus aspiration was reported in findings of this study (72).
EXPORT	Multi-centre	Export	249	In patients present with AMI, marked improvement in myocardial perfusion was reported in patients treated with primary aspiration with the Export aspiration catheter followed by direct stenting as compared with conventional stenting (73).
EXPIRA	Single-centre	Export	175	Findings of study suggested that risk of thrombus embolization is reduced with the use of thrombectomy which also preserves microvascular integrity and reducing infarct size (69).
TAPAS	Single-centre	EXPORT	1,071	Marked reduction in incidence of cardiac death at the follow-up of 1-year was reported in patients of thrombus aspiration group as compared to conventional PPCI (57).
Lipiecki et al.	Single-centre	EXPORT	44	No marked decline in infarct size or severity was reported in patients treated with thrombus aspiration with the export catheter (74).

PCI, percutaneous coronary intervention; MBG, myocardial blush grade; STR, ST-segment resolution; ECG, electrocardiogram; STEMI, ST-elevation myocardial infarction; LV, left ventricle; PPCI, primary percutaneous coronary intervention; AMI, acute myocardial infarction.

#### 6.2.1 Summary and practical aspects of thrombectomy in managing large clot burden

1. Thrombectomy as a treatment option
  • Thrombectomy aims to manage intracoronary thrombi by excluding, extracting, or dissolving them. It is particularly beneficial in cases with large thrombus burdens to prevent complications, including distal embolization, MVO, and no-reflow.
2. Manual vs. mechanical aspiration
  • Manual aspiration thrombectomy improves TIMI flow and MBG but has shown no advantages on mortality or cardiovascular events in large-scale trials and is associated with an increased risk of stroke.
  • Mechanical aspiration devices provide continuous aspiration, with better outcomes in registry data; however, they lack randomized comparisons to manual devices.
3. Current guidelines
  • Routine TA is not recommended in STEMI owing to the lack of clinical benefit and stroke risk.
  • Cases with unsuccessful balloon angioplasty or a high risk of distal embolization exists are advised for bailout thrombectomy.
4. Optimal thrombectomy technique
  • Techniques include avoiding tortuous arteries, active antegrade aspiration, deep guiding catheter positioning during withdrawal, and rigorous device flushing to prevent complications, including thrombus dislodgement or air embolization.
5. Adjunctive therapy for residual thrombus
  • IV or intracoronary GPIs and fibrinolytic agents can be used for large residual thrombus or no-reflow cases.
  • Intracoronary drug delivery ensures higher local drug concentrations but has shown mixed results in various trials.
6. Excimer laser for refractory thrombus
  • Excimer Laser Coronary Angioplasty (ELCA) may be used in refractory cases to “vaporize” the thrombus, especially in SVGs or COVID-19 related STEMI.
  • It is reserved for select cases owing to the risks of coronary rupture and higher cost.
7. Deferral of stenting
  • Deferred stenting, with prolonged antiplatelet and antithrombotic therapy followed by a staged angiography, is an option for cases with persistent thrombus to reduce the risk of no-reflow.
  • The clinical outcomes using this strategy remain inconclusive.
8. Subgroup findings
  • Meta-analyses of TA have shown no reduction in cardiovascular death, with a trend toward increased stroke incidence in patients with large thrombus burdens.
9. Integration with imaging
  • Intracoronary imaging (OCT or IVUS) helps guide thrombectomy by identifying thrombus characteristics and related complications, including stent malapposition, underexpansion, or fractures.
10. Decision pathway
  • Thrombectomy strategies should be individualized on the basis of thrombus burdens, lesion characteristics, and procedural risks. Incorporating advanced techniques, such as thrombectomy, pharmacotherapy, or excimer laser, may optimize outcomes in difficult cases.

New suction devices, including the Penumbra Indigo System, provide considerable advancements over manual aspiration for PPCI, particularly in cases with large thrombus burdens.

### 6.2 Mechanical thrombectomy devices

Depending on the potential to undergo fragmentation of the atherosclerotic thrombus material before aspiration, mechanical l thrombectomy devices exhibit distinct operational mechanism. The AngioJet, X-Sizer, and Rinsipratory system are associated with a significant risk of active thrombus fragmentation.

(a) AngioJet rheolytic thrombectomy

AngioJet rheolytic thrombectomy is the most commonly utilized mechanical thrombectomy device in PPCI. It employs high-velocity saline jets for inducing thrombus dissolution, creating a Venturi effect at the catheter tip that generates strong suction of approximately 600 mmHg to effectively aspirate the thrombus. Three randomized trials have evaluated the clinical efficacy of the AngioJet system as an adjunct to PPCI. The first trial demonstrated its benefit in reducing the infarct size compared with standard PPCI, as assessed using STR and technetium-99 m sestamibi scintigraphy (69). However, these findings were not replicated in the larger multicenter AIMI trial, which failed to demonstrate significant differences (70).

The JETSTENT trial, which enrolled 501 patients with STEMI with visible thrombus, reported no significant differences between direct stenting and mechanical thrombectomy in terms of STR, TIMI blush grade-3, or infarct size. However, follow-up at 1 and 6 months revealed a significant reduction in MACE and improved 1-year event-free survival with the use of MT (71).

Based on the available evidence, the AngioJet system provides clinical benefits in patients with substantial thrombus burdens but does not offer considerable advantages in those with minimal thrombus burdens. For optimized outcomes, selective use of this system should be considered in cases of HTBs.

(b) X-sizer thrombectomy system

X-sizer has emerged as the user-friendly mechanical thrombectomy device with the X-Sizer thrombectomy catheter. Powered by a handheld controller, it features a helical cutter that rotates at 2,100 rpm. Once engaged, the catheter captures the thrombus using vacuum suction, directing it into the inner lumen where the distal tip's helical cutter shears the thrombus for removal.

The X-TRACT-AMI study, involving 216 patients, showed no significant improvement in TIMI III flow or MBG after using the X-Sizer device. Despite this, MACEs were avoided in 93% of patients at 30 days and 83% at 360 days. Overall, clinical outcomes remained favorable (72).

Despite these advantages, the X-Sizer catheter has notable limitations. Its rigidity restricts its use in heavily calcified or tortuous vessels. Additionally, its bulky design precludes its use in arteries smaller than 2.5 mm and in very tight lesions. The risk of coronary artery perforation is increased owing to the presence of the cutting blade, housed within the outer lumen, further limiting its routine application in PPCI (73). These drawbacks underscore the need for careful patient selection when considering the X-Sizer catheter for thrombus management.

(c) Rinspirator system

The Rinspirator system (eV3 Inc., MN) is a novel nonmanual thrombectomy device designed with three lumens:

(a) **Guidewire passage**: The first lumen allows the device to pass over a standard coronary guidewire.
(b) **Distal aspiration**: The second lumen facilitates the aspiration of thrombotic material from the vessel.
(c) **Rinsing solution infusion:** A heparinized saline solution is injected by the third lumen through perforations located proximally to the aspiration lumen. Turbulent flow is generated by this solution which rinses the wall of the vessel; detaches the thrombus adhered to the vessel wall and simultaneously evacuates the material.

This simultaneous rinsing and aspiration mechanism aims to effectively clear thrombotic obstructions. Although initial data from international registries have suggested that the Rinspirator system is safe and does not significantly increase complication rates, its clinical efficacy as an adjunct to PPCI has yet to be conclusively demonstrated (74).

### 6.3 CAT catheter indigo system

Considering the limitations of existing devices, which frequently fail to remove organized thrombus, adequately revascularize vessels with large clot burdens, or prevent distal embolization, a novel system called the Indigo™ Catheter System (Penumbra®, Alameda, CA, USA) has emerged. Commercially introduced in the United States in 2014, this system has garnered substantial attention for managing thromboembolic disease in peripheral arteries, including the visceral and upper extremities.

The Indigo system utilizes a vacuum pump that generates substantial suction (29 mmHg) to aspirate clots of varying lengths and sizes, thereby promoting effective thrombectomy. The device comprises the following three main components: the separator, pump, and aspiration catheter. Two sizes of aspiration catheters, CAT 5 and CAT 3, are primarily used. The separator facilitates clot mobilization and catheter lumen cleaning, ensuring continuous aspiration and flow restoration. This system offers a major advantage in that it eliminates the requirement for lytics, enabling rapid blood flow reestablishment.

**Figure 4:**
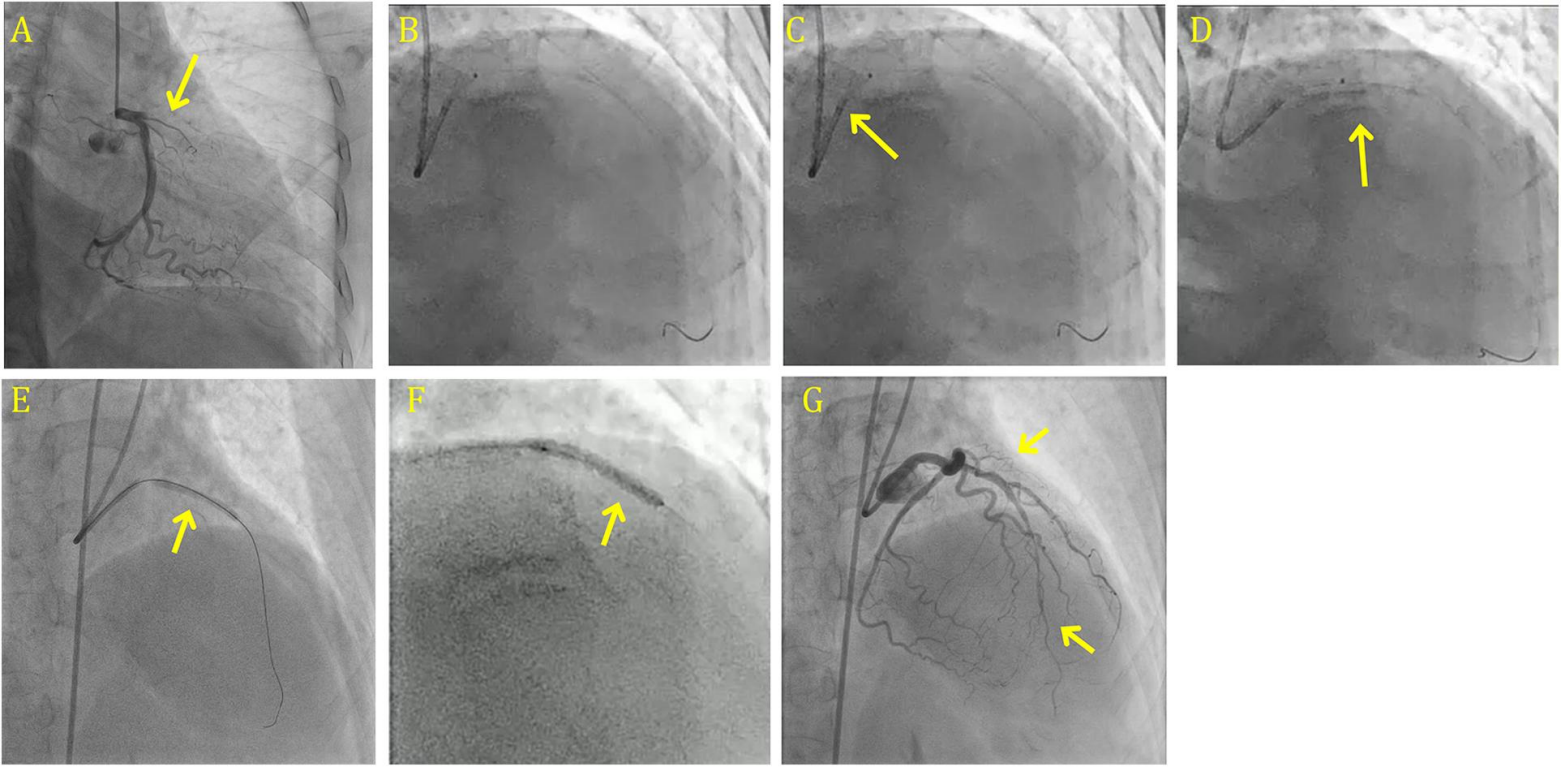
Large thrombus burden angioplasty done aided by CAT RX aspiration catheter. **(A)** CAG shows the highly thrombosed ostial LAD 100% with grade 6 clot. **(B)** CAG shows the crossing of lesion successfully with the usual workhorse wire. **(C)** CAG shows the aspiration with mechanical aspiration system- CAT RX Aspiration Catheter (Penumbra indigo system) first pass conducted in the proximal LAD. **(D)** CAG shows the aspiration second pass performed in the mid and proximal LAD. **(E)** CAG shows the deployment of 3.5 × 26 mm DES. **(F)** CAG shows the use of 3.0 × 20 mm DCB in the mid LAD. **(G)** Final results with TIMI 3 flow and expanded stent. CAG, coronary angiography; LAD, left anterior descending artery; DCB, drug coated balloon.

To evaluate its safety and clinical performance in patients with large ICT burdens, a multicenter prospective CHEETAH study was conducted. This single-arm post-market registry included 400 patients from 25 hospitals in the United States enrolled between August 2019 and December 2020. The study demonstrated that sustained mechanical aspiration before PCI using the CAT RX Aspiration Catheter was safe and yielded favorable clinical outcomes, including successful flow restoration, normal myocardial perfusion on final angiography, and high ICT removal rates. Notably, no device-related serious adverse events were reported, further supporting its safety and efficacy in this high-risk patient population (75). Large thrombus burden angioplasty done aided by CAT RX Aspiration Catheter is shown in Figure 4

### 6.4 Advantages of the penumbra and new suction devices

#### 6.4.1 Continuous aspiration with higher efficiency

Penumbra's mechanical system provides continuous controlled aspiration, which is more effective at removing large thrombi than manual devices, which rely on intermittent suction. This reduces the likelihood of incomplete thrombus removal and reduces distal embolization.

#### 6.4.2 Better handling of large thrombus burdens

Penumbra's larger luminal catheters and stronger suction capabilities are specifically designed for large thrombus burdens. These features make it a superior alternative in cases where manual aspiration may fail or be inefficient.

#### 6.4.3 Minimized operator dependency

Manual aspiration is technique-dependent and can cause variability based on the operator's experience. The Penumbra devices standardize the process, resulting in more consistent results.

#### 6.4.4 Reduced risk of stroke

Randomized trials on manual aspiration have shown an increased risk of stroke due to a dislodged thrombus or air embolization. Penumbra devices feature advanced systems that mitigate these risks by minimizing embolization into the side branches or the aorta.

#### 6.4.5 Real-time thrombus removal

Real-time visual assessment of thrombus removal during aspiration enables more informed decision-making for subsequent procedural decisions.

#### 6.4.6 Broad clinical applications

These devices can handle challenging anatomies, including tortuous vessels, which are difficult to navigate using manual aspiration catheters. They may also be beneficial in cases of complex STEMI, including thrombus in SVGs or COVID-19 related thrombotic occlusions.

### 6.5 Challenges and considerations

#### 6.5.1 Cost and availability

Devices, such as the Penumbra, are more expensive than manual aspiration, which may limit their widespread adoption in resource-constrained settings.

#### 6.5.2 Learning curve

Using these advanced systems requires training and familiarity, which may initially pose a barrier for some operators.

#### 6.5.3 Lack of large-scale randomized data

Although the Penumbra has shown promising results in registries and small studies, robust randomized trials directly comparing it with manual aspiration in PPCI remain needed.

### 6.6 Clinical implications

New suction devices, including the Penumbra, present viable alternatives to manual aspiration, particularly in cases of high-risk STEMI or when a large thrombus burden is present. Their efficiency, safety, and adaptability make them a valuable addition to the PPCI armamentarium. However, broader adoption will depend on further clinical data, cost-effectiveness, and operator experience.

### 6.7 Summary of the meta-analysis on manual versus mechanical thrombectomy (76)

#### 6.7.1 Purpose

This meta-analysis compared the efficacy of manual and mechanical thrombectomy during PCI for STEMI using direct and adjusted indirect comparisons to assess clinical and procedural outcomes.

#### 6.7.2 Data sources and inclusion

The study analyzed three trials that directly compare manual and mechanical thrombectomy (*N* = 513) and 21 RCTs that indirectly compare these strategies via a common comparator (*N* = 4,514).

#### 6.7.3 Key findings

Direct comparisons showed no significant differences in survival, reinfarction, or procedural outcomes between the two methods. Indirect meta-analysis suggested that compared with mechanical thrombectomy, manual thrombectomy reduced mortality in general populations with STEMI.

#### 6.7.4 HTB

When excluding trials with <50% intracoronary thrombus at baseline, mechanical thrombectomy demonstrated lower rates of reinfarction (*p* < 0.001) and stroke (*p* = 0.04) than manual thrombectomy.

#### 6.7.5 Procedural outcomes

Both strategies improved coronary flow (TIMI-3) and STR, with comparable effectiveness in most settings.

#### 6.7.6 Strengths of mechanical thrombectomy

In cases of HTB, mechanical devices demonstrated superiority by enabling more effective thrombus removal and reducing adverse events, including MI and stroke recurrence.

#### 6.7.7 Challenges

The limited number of studies and patients restricted the availability of direct evidence, highlighting the need for additional rigorous trials.

#### 6.7.8 Indirect methodology

The adjusted indirect meta-analysis preserved randomization, enhancing the reliability of the indirect comparisons between manual and mechanical thrombectomy.

#### 6.7.9 Implications

Mechanical thrombectomy may be preferred in patients with a HTB, whereas manual thrombectomy remains comparable in broader populations with STEMI.

#### 6.7.10 Conclusion

Mechanical thrombectomy provides specific advantages in selected patient groups; however, overall survival and procedural outcomes are largely similar between the two strategies.

### 6.8 Summary of manual vs. mechanical thrombectomy with relevant studies and imaging insights

#### 6.8.1 Comprehensive meta-analysis

This meta-analysis is the first to systematically compare manual and mechanical thrombectomy using both direct and indirect evidence. It highlights that mechanical thrombectomy provides benefits in reducing MI and stroke recurrence rates in patients with a HTB.

#### 6.8.2 Impact of the thrombus burden

HTB is a predictor of distal embolization, no-reflow, and impaired tissue perfusion, which increase the risk of MACE. Studies, such as the ATTEMPT meta-analysis (2,686 patients), reported that thrombectomy reduced MACE; however, survival benefits were limited to populations undergoing manual thrombectomy, potentially owing to selection bias.

#### 6.8.3 Procedural outcomes

Both manual and mechanical thrombectomy improved TIMI-3 flow and STR, reflecting their capacity to improve epicardial perfusion. However, mechanical thrombectomy demonstrated superior thrombus removal, as shown in the SMART Primary PCI Trial, which utilized OCT to compare thrombus burden reduction between the two strategies.

#### 6.8.4 Role of intracoronary imaging

Imaging modalities, including OCT and IVUS, are vital for thrombus burden assessment and procedural guidance. OCT was pivotal in the SMART trial, revealing that mechanical thrombectomy was more effective in removing the thrombus and improving myocardial perfusion before stenting.

#### 6.8.5 Mechanisms of the mechanical devices

Devices, including the ANJOJET catheter utilize high-velocity saline jets (Bernoulli effect) to fragment and evacuate thrombus, achieving better removal than manual aspiration. This feature is particularly beneficial in patients with visible and large thrombi.

#### 6.8.6 Manual thrombectomy limitations

Manual aspiration is less effective for large thrombi and may cause microembolization, increasing the risk of MI recurrence, particularly in patients with a HTB. This risk was highlighted in the TREAT-MI trial, which, despite limitations, confirmed the challenges of manual thrombectomy in larger thrombi.

#### 6.8.7 Clinical insights from studies

**TAPAS trial** showed that manual thrombectomy improved surrogate reperfusion markers but emphasized the need for alternative strategies in cases of high-risk thrombus. **AIMI trial** Reported increased stroke rates with manual thrombectomy in patients with STEMI, highlighting its limitations in specific populations.

#### 6.8.8 Thrombus burden and stent outcomes

HTB may cause late stent malapposition, increasing the risk of stent thrombosis and MI recurrence as the thrombus dissolves. By achieving more complete thrombus removal, mechanical thrombectomy mitigates this risk.

#### 6.8.9 Future research directions

To refine treatment strategies, trials integrating thrombus burden assessment via OCT/IVUS and comparing manual vs. mechanical thrombectomy in high-risk populations are warranted.

#### 6.8.10 Conclusion

Imaging-guided mechanical thrombectomy demonstrates clear benefits in patients with a large thrombus burden, whereas manual thrombectomy remains effective in cases with lower thrombus burdens. The choice of strategy should integrate angiographic findings and intracoronary imaging for optimal outcomes.

## 7 ELCA

The CVX-300 cardiovascular excimer laser system, developed by Spectranetics (now part of Philips), utilizes xenon chloride as the active medium to emit high-energy ultraviolet light at a 308 nm wavelength (77). This technology enables the precise atherosclerotic plaque and thrombus ablation with minimal thermal injury to the surrounding tissues. The system comprises a laser generator and specialized catheters designed for coronary interventions (78).

**Figure 5:**
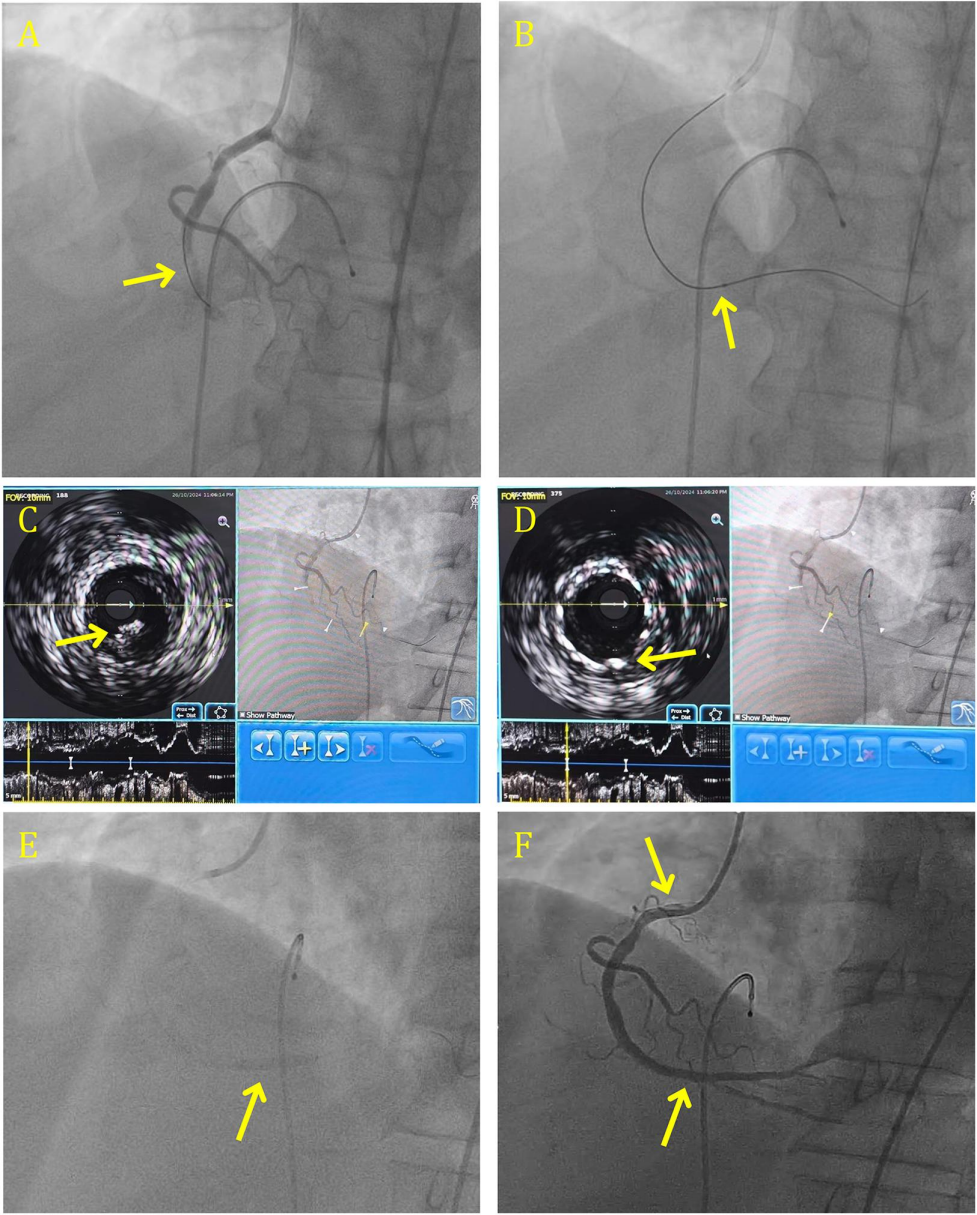
Large thrombus burden angioplasty done aided by ELCA. **(A)** Pre-PCI CAG shows mid RCA 100% occluded with grade 6 clot & TPI. **(B)** CAG shows the successful crossing of lesion with ELCA (Pulses 40–80 in 3 rows). **(C)** IVUS post ELCA still visible clot. **(D)** IVUS post stent fully expanded. **(E)** CAG shows the persistent clot in the distal PLV branch, prompting direct stenting with a 3.0 × 20 mm DES in the PLV. **(F)** Good final result with TIMI 3 flow filling distal RCA, PLV & PDA. PCI, percutaneous coronary angiography; CAG, coronary angiography; RCA, right coronary artery; TPI, temporary pacemaker implantation; ELCA, excimer laser coronary angioplasty; IVUS, intravascular ultrasound; PLV, posterior left ventricular; PDA, patient ductus arteriosus.

The CARMEL multicenter trial (The registry of Acute Revascularization in Myocardial Infarction with Excimer Laser multicenter) registry evaluated the safety and efficacy of ELCA in patients with AMI. The study enrolled 151 patients with AMI, 65% of patients were present with large thrombus burden in the culprit artery. Following ELCA, improvements were observed in the TIMI flow grades, indicating enhanced reperfusion (67). Despite these promising results, the routine use of the CVX-300 system in PPCI remains limited owing to various factors, including the need for specialized equipment, operator expertise, and considerations regarding cost-effectiveness. To establish the definitive role of the excimer laser technology in PPCI settings, further large-scale RCTs are necessary. Large thrombus burden angioplasty done aided by ELCA is shown in Figure 5.

## 8 Embolic protection devices (EPDs)

EPDs have been developed for reduce the risk of distal embolization during interventions in SVGs and carotid arteries (79). Although their use in SVG interventions has demonstrated significant clinical benefits, similar advantages have not been replicated in STEMI management during PPCI.

EPDs are categorized into the following three types on the basis of their operational mechanism (80):

1. **Distal filters**: these devices capture embolic debris downstream from the intervention site, facilitating blood flow while trapping larger debris.
2. **Distal occlusion aspiration devices**: these temporarily occlude the vessel distal to the lesion, preventing embolization, while an aspiration mechanism removes the trapped debris.
3. **Proximal occlusion aspiration devices**: these devices occlude the vessel proximally, halting antegrade blood flow and embolization during the procedure, with debris removed via aspiration.

Despite their theoretical advantages, the routine use of EPDs in STEMI has not been supported by evidence, and their application remains limited to specific contexts, including SVG and carotid interventions. Further studies may clarify their role in broader clinical settings.

### 8.1 Aspiration devices with distal occlusion

A flexible tube with occlusion balloon, which can be inflated is a major characteristics feature of distal occlusion aspiration devices. In this procedure, several centimetres distal to the lesion, the balloon was passed and inflated using a carbon dioxide-filled syringe to block antegrade blood flow. This strategy prevents distal embolization during PCI. However, it has significant drawbacks, including the inability to fully visualize the lesion owing to complete flow blockage and the risk of thrombogenesis and slow-flow states.

EMERALD trial (The Exploring the Mechanism of Plaque Rupture in ACS Using Aspiration of Liberated Debris in Acute MI with GuardWire Plus System trial) evaluated the safety and efficacy of adjunctive PercoSurge (a distal occlusion device) compared with conventional PCI (68). Device safety is demonstrated by the study, but it did not demonstrate the clinical outcomes in terms of infarct size reduction. These findings indicate that although distal occlusion devices may be safe, their routine use in PCI for STEMI lacks robust clinical impact and remains unsupported for broad adoption (68).

### 8.2 Clot management- special situations

#### 8.2.1 Managing stent thrombosis

##### 8.2.1.1 Incidence and presentation

Stent thrombosis occurs in approximately 1% of patients within the first 2-years following PCI, with most cases presenting as STEMI. Premature discontinuation of DAPT and stent-related issues, including underexpansion, fracture, malapposition, edge dissection, stent gap, residual uncovered plaque, and undersizing, are common causes of stent thrombosis.

##### 8.2.1.2 Role of intracoronary imaging

Intracoronary imaging with OCT or IVUS is crucial to identify the cause of stent thrombosis. OCT offers higher resolution and better visualization of vascular issues, including uncovered struts, malapposition, stent fracture, and neo-atherosclerosis.

##### 8.2.1.3 Mechanism-Based intervention

Underexpansion or malapposition: treated with high-pressure balloon inflation using an appropriately sized balloon based on imaging findings. Stent fracture, edge dissections, or neo-atherosclerosis: typically managed with the placement of a second DES.

##### 8.2.1.4 Large thrombus burden management

Stent thrombosis frequently presents with a significant thrombus burden, requiring careful integration of thrombus management strategies, including thrombectomy, pharmacotherapy, or deferred stenting as appropriate.

##### 8.2.1.5 Prevention and long-term management

Key preventive measures include ensuring optimal stent deployment and adherence to DAPT protocols. The risk of future stent thrombosis can be mitigated by regular follow-ups and intracoronary imaging during complex interventions.

**Figure 6:**
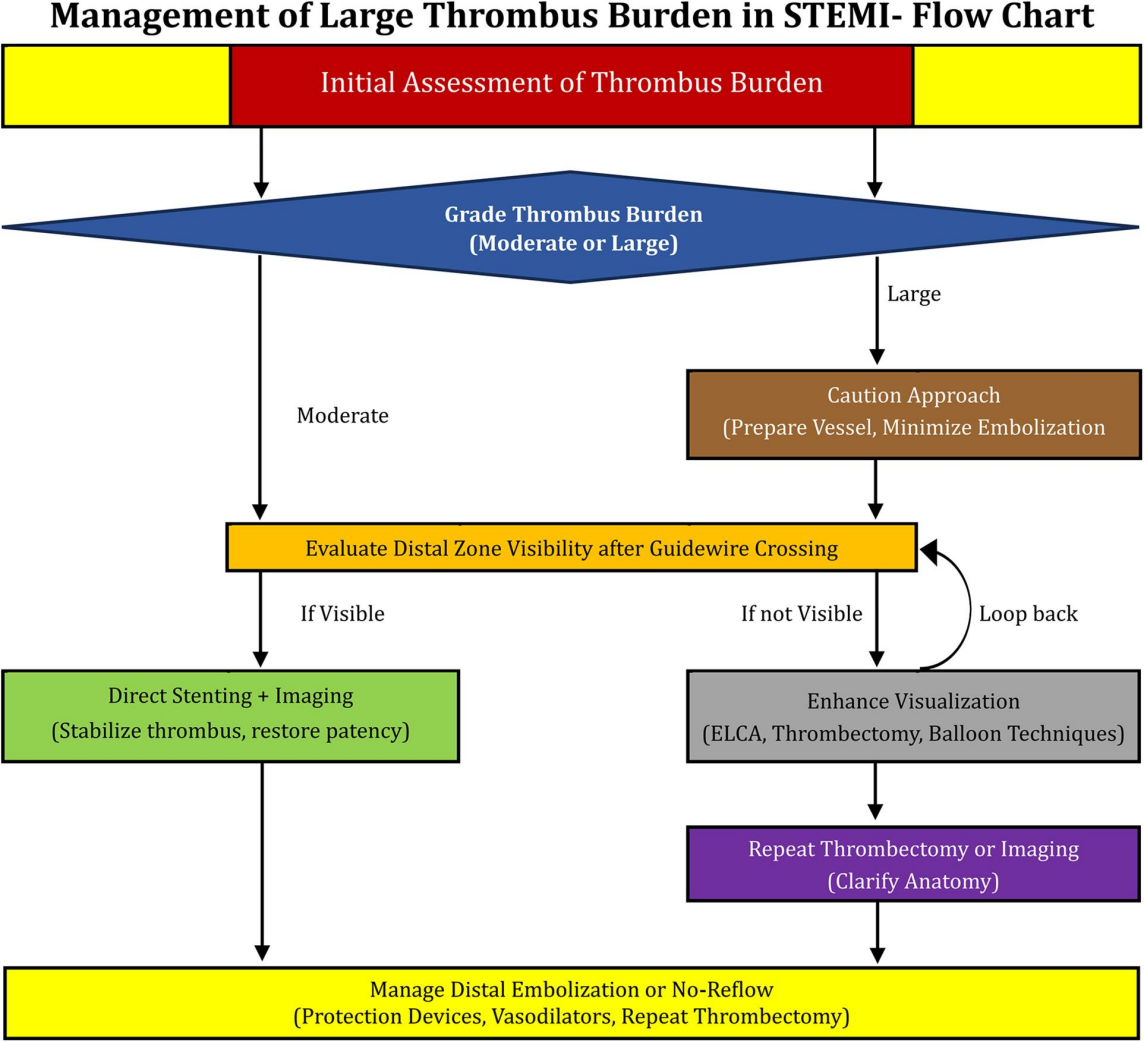
The management protocol of approach to large thrombus burden in STEMI.

Ten commandments on intracoronary thrombus management are demonstrated in Table 5. The management protocol of approach to large thrombus burden in STEMI is shown in Figure 6.

**Table 5:** Ten commandments on intracoronary thrombus management.

1. **Impact of the intracoronary thrombus** • Intracoronary thrombus is associated with complications, including distal embolization, MVO, no-reflow, side-branch compromise, and late stent malapposition. • Proper classification of the thrombus using the TIMI thrombus grade is essential for guiding PCI strategies.
2. **Thrombus classification** • The thrombus is categorized as either large (TIMI grades 4–5) or small/no thrombus burden (TIMI grades 0–3). • Thrombotic grade assessment should be performed following guide wire placement owing to rapid changes following lesion crossing.
3. **Role of direct stenting** • Direct stenting can help stabilize the thrombus and reduce procedural complications by limiting vessel manipulation.
4. **Use of direct stenting** • It may be considered in cases with a small thrombus burden or even in moderate to large thrombus burden if distal landing zone is visible or after it is made visible.
5. **TA strategies** • One of the strategies for large thrombus burdens include manual and mechanical aspiration thrombectomy. • Owing to the lack of mortality benefit and potential stroke risk, routine manual aspiration is no longer recommended; however, selective use as a bailout remains viable.
6. **Optimal thrombectomy techniques** • Effective aspiration encompasses avoiding tortuous arteries, maintaining active aspiration, and proper catheter management for preventing thrombus dislodgement or air embolization. • To ensure complete thrombus removal, rigorous device flushing is crucial.
7. **Adjunctive pharmacotherapy** • Intracoronary or IV GPIs and fibrinolytics may be used for persistent thrombus or no-reflow cases. • Intracoronary GPIs have demonstrated benefits in improving reperfusion markers but lacks long-term outcome benefits.
8. **Excimer laser for refractory thrombus** • Excimer laser angioplasty is a specialized alternative for cases of refractory thrombus, particularly in SVGs or COVID-19–related STEMI. • It should be used cautiously owing to the risks of coronary rupture and cost considerations • especially useful in large thrombus burden in ecstatic or large arteries.
9. **Decision pathway and guidelines 1** • Management decisions should incorporate thrombus classification, the potential role of direct stenting, thrombectomy techniques, and adjunctive therapies.
10. **Decision pathway and guidelines 2** • The current guidelines recommend against routine aspiration in STEMI but support tailored strategies, including direct stenting and advanced methods, for high-risk thrombus cases.

^†^MVO: Microvascular Obstruction; TIMI; Thrombolysis in Myocardial Infarction; PCI: Percutaneous Coronary Intervention; GPI: Glycoprotein; SVG: Saphenous Vein Graft; STEMI: ST-Elevation Myocardial Infarction.

### 8.3 Summary and practical aspects of thrombectomy in managing large clot burden

1. Thrombectomy as a treatment option
  1.1 Thrombectomy aims to manage intracoronary thrombi by excluding, extracting, or dissolving them. It is particularly beneficial in cases with large thrombus burdens to prevent complications, including distal embolization, MVO, and no-reflow.
2. Manual vs. mechanical aspiration
  2.1 Manual aspiration thrombectomy improves TIMI flow and MBG but has shown no advantages on mortality or cardiovascular events in large-scale trials and is associated with an increased risk of stroke.
  2.2 Mechanical aspiration devices provide continuous aspiration, with better outcomes in registry data; however, they lack randomized comparisons to manual devices.
3. Current guidelines
  3.1 Routine TA is not recommended in STEMI owing to the lack of clinical benefit and stroke risk.
  3.2 Cases with unsuccessful balloon angioplasty or a high risk of distal embolization exists are advised for bailout thrombectomy.
4. Optimal thrombectomy technique
  4.1 Techniques include avoiding tortuous arteries, active antegrade aspiration, deep guiding catheter positioning during withdrawal, and rigorous device flushing to prevent complications, including thrombus dislodgement or air embolization.
5. Adjunctive therapy for residual thrombus
  5.1 IV or intracoronary GPIs and fibrinolytic agents can be used for large residual thrombus or no-reflow cases.
  5.2 Intracoronary drug delivery ensures higher local drug concentrations but has shown mixed results in various trials.
6. Excimer laser for refractory thrombus
  6.1 Excimer Laser Coronary Angioplasty (ELCA) may be used in refractory cases to “vaporize” the thrombus, especially in SVGs or COVID-19 related STEMI.
  6.2 It is reserved for select cases owing to the risks of coronary rupture and higher cost.
7. Deferral of stenting
  7.1 Deferred stenting, with prolonged antiplatelet and antithrombotic therapy followed by a staged angiography, is an option for cases with persistent thrombus to reduce the risk of no-reflow.
  7.2 The clinical outcomes using this strategy remain inconclusive.
8. Subgroup findings
  8.1 Meta-analyses of TA have shown no reduction in cardiovascular death, with a trend toward increased stroke incidence in patients with large thrombus burdens.
9. Integration with imaging
  9.1 Intracoronary imaging (OCT or IVUS) helps guide thrombectomy by identifying thrombus characteristics and related complications, including stent malapposition, underexpansion, or fractures.
10. Decision pathway
  10.1 Thrombectomy strategies should be individualized on the basis of thrombus burdens, lesion characteristics, and procedural risks. Incorporating advanced techniques, such as thrombectomy, pharmacotherapy, or excimer laser, may optimize outcomes in difficult cases.

New suction devices, including the Penumbra Indigo System, provide considerable advancements over manual aspiration for PPCI, particularly in cases with large thrombus burdens.

## 9 Conclusion

Managing ICT during PCI, especially in STEMI with a HTB, remains a significant challenge owing to complications, including no/slow-reflow and distal embolization. Although no universal strategy exists, an individualized approach based on thrombus assessment and operator expertise is significant. Mechanical thrombectomy and aspiration, pharmacological interventions with potent antiplatelet and anticoagulant agents, and targeted use of stenting techniques such as direct or deferred stenting represent key strategies. Intracoronary thrombolysis provides advantages in enhancing myocardial perfusion, whereas newer-generation DESs have demonstrated improved outcomes. Although mesh-covered stents hold promise, more clinical trials are required to confirm their role in optimizing thrombus management and improving long-term outcomes.
